# E-Cigarette or Vaping Product Use-Associated Lung Injury (EVALI) Mimicking COVID-19 Disease

**DOI:** 10.1155/2020/8821289

**Published:** 2020-11-01

**Authors:** Jose A. Rodriguez, Alejandra A. Roa, Juan C. Lemos-Ramirez

**Affiliations:** ^1^Memorial Hospital West, Memorial Healthcare System, Pembroke Pines, FL, USA; ^2^Division of Infectious Disease, Memorial Regional Hospital, Memorial Healthcare System, Hollywood, FL, USA

## Abstract

**Background:**

Coronavirus 2 (SARS-CoV-2) is the virus associated with the coronavirus disease (COVID-19) causing a pandemic worldwide in 2020. There are other noninfectious diseases that can present exactly as COVID-19, and the management and approach are completely different, hence the importance of understanding and having a wide differential in patients presenting with similar characteristics. *Case Report*. A 23-year-old male, with a history of childhood asthma, presented to the Emergency Department in a hospital in south Florida in the USA with complaints of a 2-day duration of subjective fever, chills, dry cough, dyspnea, and myalgia. His vital signs were blood pressure 135/65 mmHg, temperature 39°C, pulse 134 bpm, respiratory rate 22 breaths per minute, and saturation of oxygen 96% in room air. Laboratory analysis was significant for white blood cells 15.3 × 10^3^/*μ*L, ALT 69 U/L, AST 66 U/L, ferritin 375.6 ng/mL, C-reactive protein 27.70 mg/dL, and procalcitonin 1.43 ng/mL. A respiratory pathogen panel (RPP) and a SARS-CoV-2 test were both negative. The patient was given empiric antibiotic treatment and hydroxychloroquine. Two more tests for SARS-CoV-2 were negative, and the patient reported that he smoked marijuana through an e-cigarette. The patient was started on high-dose steroids, and symptoms improved.

**Conclusion:**

COVID-19 is an emergent lung disease that is affecting the population worldwide; many other noninfectious diseases can mimic its presentations and laboratory characteristics; the importance of having a broad differential diagnosis especially in causing confusion during pandemic times is valuable in the management of patients with such presentations, such as EVALI, and glucocorticoids will be indicated in this circumstances.

## 1. Introduction

Coronavirus 2 (SARS-CoV-2) is the virus associated with the coronavirus disease (COVID-19) causing a pandemic with multiple morbidities worldwide (1). There are other noninfectious diseases that can present as COVID-19, and the management and approach are completely different, hence the importance of understanding and having a wide differential in patients presenting with similar characteristics. In this case, we present a young male with clinical and laboratory characteristics of a COVID-19 patient without any positive tests documented.

## 2. Case Report

**Figure 1:**
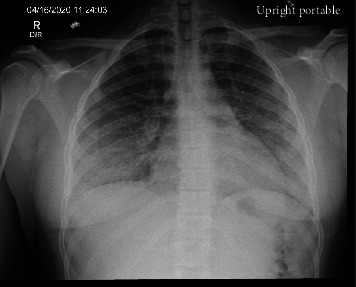
Chest X-ray: diffuse bilateral lung infiltrates with basilar predominance.

A 23-year-old male, with a history of childhood asthma, presented to the Emergency Department (ED) in a hospital in South Florida in the USA with complaints of a 2-day duration of subjective fever, chills, dry cough, dyspnea, and myalgia. He denied nausea, vomiting, diarrhea, abdominal pain, sputum production, and known contacts with COVID-19-positive persons. There are no recent travels. He admitted taking NSAIDs for his symptoms without any improvements. His vital signs at presentation were blood pressure 135/65 mmHg, temperature 39°C, pulse 134 bpm, respiratory rate 22 breaths per minute, and SatO_2_ 96% in room air. On physical examination, he was febrile to touch, tachycardic, alert, and in no respiratory distress, has no wheezing, and has no rales. Laboratory analysis was significant for white blood cells 15.3 × 10^3^/*μ*L, neutrophils 86.8%, lymphocytes 7.9%, hemoglobin 13.3 g/dL, hematocrit 38.7%, platelets 290 × 10^3^/*μ*L, BUN 8 mg/dL, creatinine 0.88 mg/dL, albumin 2.8 g/dL, ALT 69 U/L, AST 66 U/L, ferritin 375.6 ng/mL, C-reactive protein (CRP) 27.70 mg/dL, and procalcitonin 1.43 ng/mL, and urinalysis is unremarkable. A respiratory pathogen panel (RPP) (GenMark Diagnostics, Carlsbad, CA) and a SARS-CoV-2 test (CDC 2019-Novel Coronavirus Real-Time RT-PCR Diagnostic Panel for use under a Food and Drug Administration's Emergency Use authorization approved 2/4/2020) were performed on nasopharyngeal swab specimens of both nostrils coming back both negative. A chest X-ray was obtained (Figure 1) showing bilateral interstitial infiltrates; blood cultures were sent. Empiric ceftriaxone 1 gm IV daily, doxycycline 100 mg orally twice a day, 1 L of normal saline, and acetaminophen 1 gm orally were given at the ED. Due to the high clinical suspicion, the patient was placed in respiratory isolation in a single negative pressure ward.

**Figure 2:**
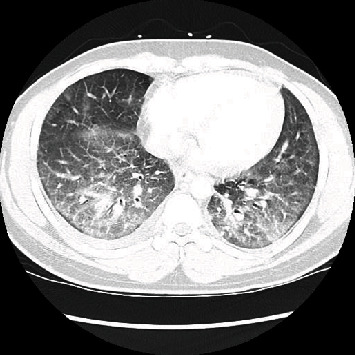
Chest CT: bilateral mixed interstitial and ground-glass infiltrates. Lower lobe predominance. Small right pleural effusion.

The following day, the patient started having several episodes of liquid stools, still had high-grade fever (39.1°C) with O_2_ saturation dropped to the 80s when ambulating, and had to be placed on nasal cannula 2 L. A SARS-CoV-2 test was repeated with a negative result. Liver transaminases, D-dimer, and WBC were improving, and LDH was 275 U/L. An infectious disease consult was placed. An HIV test, IL6, and urinary streptococcal antigen were ordered, and the patient was started on hydroxychloroquine 200 mg PO bid due to the high clinical suspicion for COVID-19. CT chest with IV contrast was obtained (Figure 2), and pulmonary embolism was ruled out. Blood and urine cultures were negative. Urine toxicology was positive for marijuana and opioids. On further interrogation, the patient reported he smokes marijuana through the use of an e-cigarette 3 times a week for the last 2 years. On the 3^rd^ day of hospitalization, a third SARS CoV-2 test was reordered through an endotracheal sample with negative results; hydroxychloroquine was discontinued, IL6 resulted 21.28 pg/mL, Streptococcus pneumonia antigens in the urine were not detected, and HIV antigen is nonreactive. He was started on methylprednisolone 60 mg IV q6h. 32 hours after initiation of high-dose steroids, the patient was weaned off from oxygen supplementation, diarrhea resolved, cough improved, transaminases improved, and he was discharged on the prednisone tapering dosage.

## 3. Discussion

The respiratory effects of e-cigarettes are still undergoing investigation; current studies do show measurable adverse biologic effects on organ and cellular health in humans, animals, and in vitro (2). E-cigarette or vaping product use-associated lung injury (EVALI) is a diagnosis of exclusion. The most common clinical characteristics of EVALI include dyspnea, cough, chest pain, fever, chills, nausea, vomiting, and diarrhea (3–5). As published in multiple studies, these are similar to the symptoms COVID-19 patients present with (6, 7). Also, as in COVID-19, EVALI is more prevalent and manifests with worse outcomes in patients with underlying chronic conditions including obesity (52%) and cardiac (47%) and respiratory (23%) diseases as presented in a national study (8). Chest CT findings in EVALI most commonly show a pattern of acute lung injury on the spectrum of organizing pneumonia and diffuse alveolar damage (9). A systematic review (Jonas and Raj (10)) includes data on 216 patient cases spanning 41 articles of parenchymal lung injury attributable to vaping, describing various imaging patterns which can aid the clinician in evaluation of a patient with suspected EVALI; a combination of ground-glass opacities, consolidations, and nodular opacities in various distribution patterns was seen; they describe in some cases features suggestive of acute hypersensitivity pneumonitis, with upper and midlung predominant ground-glass opacity and ill-defined centrilobular ground-glass nodules and in one case similar to the imaging found in sarcoidosis, including bilateral clusters of micronodules and ground-glass opacities and mild enlargement of mediastinal lymph nodes (10).

Elevated inflammatory and coagulation markers such as CRP, erythrocyte sedimentation rate (ESR), international normalized ratio (INR), procalcitonin, and IL-6 have been documented in patients with EVALI, mainly produced by the excessive production of reactive oxygen species, inflammatory cytokines, and chemokines by the e-cigarette vapor (11–13). The same biomarkers have been associated in patients with COVID-19; in this circumstance, it is invariable to discriminate between these two different entities just by laboratory markers. The diagnosis of COVID-19 relies on detection of SARS-Cov-2 by PCR methods in nasopharyngeal and endotracheal swab specimens, with positive rates of 60-90%, respectively (14, 15). Our patient tested 3 times negative for it, making it less likely for his symptoms to be attributed to this disease and more in favor of EVALI.

To note, it is also important to keep in mind that EVALI can be a predisposing factor by itself for the increased risk of susceptibility of acquiring pneumonia and COVID-19 infection, The role of vaping and virus susceptibility is yet to be determined by the lack of large power studies; however, vaping can increase the virulence and inflammatory potential of several lung pathogens (16).

The treatment for EVALI other than discontinuing the offending agent has not been well established; in the hospital setting in patients with respiratory distress, systemic corticosteroids such as methylprednisolone (40-60 mg IV every 4-6 hours) for 2-4 days with oral tapering have been used with improvement seen 24-72 hours after initial dosing, but the efficacy has not yet been established (3, 4, 17).

## 4. Conclusion

COVID-19 is an emergent lung disease that is affecting most of the population worldwide; many other noninfectious diseases can mimic its presentations and laboratory characteristics; the importance of having a broad differential diagnosis especially in causing confusion during pandemic times is valuable in the management of patients with such presentations, such as EVALI, and glucocorticoids will be indicated in this circumstances.

## Data Availability

Data is available on request.
